# Association of Abnormal Pap Smear with Occult Cervical Stromal Invasion in Patients with Endometrial Cancer

**DOI:** 10.31557/APJCP.2019.20.9.2847

**Published:** 2019

**Authors:** Kewalin Khumthong, Apiwat Aue-Aungkul, Pilaiwan Kleebkaow, Bandit Chumworathayi, Amornrat Temtanakitpaisan, Wilasinee Nhokaew

**Affiliations:** *Department of Obstetrics and Gynaecology, Faculty of Medicine, Khon Kaen University, Khon Kean, Thailand. *

**Keywords:** Pap smear, endometrial cancer, cervical stromal invasion, risk factors

## Abstract

**Objective:**

The purpose of this study was to determine the association between abnormal preoperative Pap smear results and occult cervical stromal invasion in endometrial cancer patients.

**Methods:**

Medical records were reviewed of patients with endometrial cancer who had undergone surgical staging at Srinagarind Hospital. Patients with gross cervical involvement, with an unsatisfactory Pap smear, without available Pap smear results, with no cervical intraepithelial lesion/invasive cervical cancer, or who had previously undergone pelvic radiation therapy were excluded. The patients were assigned to one of two groups according their Pap smear results (negative and epithelial cell abnormalities). Logistic regression was used to determine the independent association between an abnormal Pap smear and the risk of cervical stromal invasion.

**Results:**

All cervical smears in this study were performed as conventional Pap smears. Smears were abnormal in 50 (21.0%) of the 238 patients enrolled and normal in the remaining 188 (79.0%). The types of Pap smear abnormalities included adenocarcinoma (n=22); atypical endometrial cells (n=2); atypical glandular cells (n=17); high-grade squamous intraepithelial lesions (n=4); atypical squamous cells, cannot exclude high-grade squamous intraepithelial lesions (n=2); and atypical squamous cells of undetermined significance (n=3). After controlling for type of endometrial cancer, abnormal Pap smear results were found to be a significant independent factor that indicated cervical stromal invasion (adjusted OR 2.65; 95% CI 1.35 to 5.21).

**Conclusion:**

Endometrial cancer patients with abnormal Pap smears were strongly and independently associated with histopathologically diagnosed cervical stromal invasion.

## Introduction

About 382,000 new endometrium cancer cases and nearly 90,000 deaths worldwide were estimated to have arisen in 2018 (Bray et al., 2018). Moreover, the Surveillance, Epidemiology, and End Result (SEER) program revealed that the incidence of endometrial cancer increased 2.5% annually, with a 10% increase from 2006 to 2012 (Constantine et al., 2019). Surgical staging is a part of initial endometrial cancer treatment (Burke et al., 2014). The guidelines for surgical staging of endometrial cancer (including total hysterectomy, bilateral salpingo-oophorectomy, and bilateral pelvic and para-aortic lymph node dissection), were revised by the International Federation of Gynecology and Obstetrics (FIGO) Committee in 2009 (Abu-Rustum et al., 2011). Strong histopathologic factors that indicate a high risk of recurrence or death include FIGO staging, non-endometrioid histology, lymphovascular space invasion, and cervical stromal invasion (Amant et al., 2018). 

**Table 1:** Clinical Characteristics of Women with Endometrial Cancer by Cervical Stromal Invasion Status

	Cervical stromal invasion (n =61)	No cervical stromal invasion (n =177)	P-value†
Age (mean+SD)	58.49+8.84	58.03+8.87	0.73
Parity (number, %)			
Nulliparous	11 (18.0)	39 (22.1)	0.51
Multiparous	50 (82.0)	138 (77.9)	
Menopausal (number, %)			
Premenopausal	16 (26.2)	41 (23.2)	0.06
Postmenopausal	45 (73.8)	136 (76.8)	
Body mass index (number, %)			
Lower than 30	54 (88.5)	155 (87.6)	0.84
30 or higher	7 (11.5)	22 (12.4)	
Type of endometrial cancer (number, %)			
Type I	33 (54.1)	132 (74.6)	0.003
Type II	28 (45.9)	45 (25.4)	
Pap smear (number, %)			
Normal	39 (63.9)	149 (84.2)	0.001
Abnormal	22 (36.1)	28 (15.8)	

Type I, endometrioid adenocarcinoma grades 1 and 2; type II, endometrioid adenocarcinoma grade 3, clear cell carcinoma, serous carcinoma, and carcinosarcoma; †, Pearson Chi-square, two-sided, P-value<0.05.

**Table 2:** Multivariate Analysis: Factors Associated with Cervical Stromal Invasion in Endometrial Cancer

	N	Presence of cervical stromal invasion (n, %)	adjusted Odds ratio (95% confident interval)
Pap smear			
Abnormal	50	22 (44.00)	2.65 (1.35-5.21)
Normal	188	39 (20.74)	reference
Type of endometrial cancer			
Type II	73	28 (38.36)	2.33 (1.24-4.41)
Type I	165	33 (20.00)	reference

Type I, endometrioid adenocarcinoma grades 1 and 2; type II, endometrioid adenocarcinoma grade 3, clear cell carcinoma, serous carcinoma, and carcinosarcoma.

Cervical stromal invasion is one factor that can decrease recurrence-free and overall survival in patients with endometrial cancer (Kwon et al., 2009; Ferriss et al., 2010). In endometrial cancer patients with cervical stromal involvement, adjuvant radiation (brachytherapy and/or external beam pelvic radiation) after surgery was required but five-year overall survival were still low (Frandsen et al., 2010; Orezzoli et al., 2009; Mariani et al., 2001). Previous studies have found that aggressive surgery, such as radical hysterectomy (which has replaced simple hysterectomy as the preferred method of surgical staging), results in better prognoses in endometrial cancer with cervical stromal invasion than those without cervical stromal invasion (Orezzoli et al., 2009; Mariani et al., 2001). Conversely, Takano et al (2013) reported no survival benefit from radical hysterectomy in patients with suspected grossly cervical involvement. 

A previous study found tumors involving cervical stroma in approximately 10% of patients with endometrial cancer after surgical staging (Gao et al., 2018). A preoperative diagnosis of cervical stromal invasion may be necessary before decisions can be made regarding type of hysterectomy (simple versus radical), use of preoperative radiation therapy, and role of omitted lymphadenectomy during surgical staging. A magnetic resonance imaging (MRI) scan is the most accurate preoperative method for assessing cervical stromal involvement (Amant et al., 2018; Haldorsen and Salvesen, 2016). However, the cost of a routine preoperative MRI scan is prohibitive for many endometrial cancer patients.

The Papanicolaou (Pap) smear is well known as a useful tool for detecting significant cervical abnormalities in invasive cervical cancer. It is simple, inexpensive, and the required equipment is available at general hospitals. Previous studies have reported that 38-51% of endometrial cancer patients had abnormal Pap smear results and that abnormal cervical cytology was correlated with tumor size, tumor type, depth of invasion, presence of cervical involvement, and presence of lymphovascular invasion (Lai et al., 2015; Serdy et al., 2016). 

The aim of this study was to examine the association between abnormal Pap smears and cervical stromal invasion in patients with endometrial cancer in order assist physicians in deciding on appropriate management strategies.

## Materials and Methods

This cross-sectional study received approval by the Khon Kaen University Ethics Committee in Human Research (HE611482). We reviewed the medical records of patients diagnosed with endometrial cancer who underwent surgical staging at Srinagarind Hospital between January 2010 and August 2018. The inclusion criteria were: (1) pathologically confirmed endometrial cancer and (2) Pap smear taken within six months prior to surgical staging. The exclusion criteria were: (1) suspected or gross cervical involvement, (2) unsatisfactory Pap test, (3) history of pelvic radiation therapy, (4) synchronous high-grade cervical intraepithelial lesion/invasive cervical cancer, and (5) unknown final histopathology or incomplete data. 

Data included baseline characteristics, types of Pap smear abnormality, operative procedures, and detailed histopathologic results such as type of endometrial cancer, cervical stromal invasion, and FIGO staging. In this study, a dualistic classification of endometrial cancer was used (type I and type II). Type I included endometrioid adenocarcinoma grades 1and 2, and type II consisted of endometrioid adenocarcinoma grade 3, clear cell carcinoma, serous carcinoma, and carcinosarcoma (Suarez et al., 2016). 

The following were considered abnormal Pap smear results: (1) squamous cell abnormalities involving atypical squamous cells of undetermined significance (ASC-US), atypical squamous cells cannot exclude HSIL (ASC-H), low grade squamous intraepithelial lesion (LSIL), and high grade squamous intraepithelial lesions (HSIL); (2) glandular cell abnormalities containing atypical endocervical cells, atypical endometrial cells, atypical glandular cells, endocervical adenocarcinoma in situ (AIS), or adenocarcinoma; and (3) presence of abnormal endometrial cells. A single gynecologic pathologist (PK) examined all surgical specimens. All cases included in the analysis received an independent and blinded pathologic review. Staging of endometrial cancer patients was based on FIGO classification. 

In this study, each uterine cervix specimen was dissected and placed on four to eight slides for examination. Occult cervical stromal invasion was defined if at least one of following criteria was met: the cancer foci were bounded on two sides by normal endocervical glands (proximally and distally), the cancer foci underlied normal ectocervix or endocervical glands, or irregular infiltration into stroma deep to the tumor or a stromal response at the pushing edge of the lesion in patients with exophytic tumors protruding through the endocervical canal (Soslow, 2016). 

Descriptive statistics were used to analyze demographic baseline characteristics. Categorical variables were expressed as percentages. The Chi-square and Fisher’s exact test were used whenever appropriate to compare the two groups. A P-value < .05 was considered statistically significant. Logistic regression was used as a multivariate analysis to determine the independent connection between Pap smear results and the risk of cervical stromal invasion. An odds ratio with a 95% confidence interval (CI), which did not include unity, was considered statistically significant. All statistical analysis was performed using SPSS version 17 (SPSS Inc., Chicago, Ill., USA).

## Results

During the study period, 238 endometrial cancer patients who met the inclusion criteria were evaluated. The mean age of the patients was 58.1 years (range, 31-81 years). One hundred eighty-one (76.1%) of the patients were postmenopausal and 50 (21.0%) were nulliparous. All cervical smears in this study were performed as conventional Pap smears. Median time between Pap smear and surgical staging was 2 months (range 2-5 months). There were 50 patients with abnormal Pap smears, including adenocarcinoma (n=22), atypical endometrial cells (n=2), atypical glandular cells (n=17), HSIL (n=4), ASC-H (n=2), and ASC-US (n=3). The remaining of 188 patients had normal cervical cytology results. All patients in this study underwent simple hysterectomy with bilateral salpingo-oophorectomy with or without pelvic/para-aortic lymphadenectomy. Cervical stromal invasion was histopathologically diagnosed after surgical staging in 61 (25.1%) cases. Seventy-three patients (30.6%) were noted as having type II endometrial cancer. The patients in this study were FIGO stage IA (n=101), IB (n=66), II (n=26), IIIA (n=19), IIIB (n=2), IIIC (n=34) and IVB (n=11). Table 1 shows the demographic characteristics of the study participants. 

Abnormal Pap smear results were reported in 21.0% of cases (95%CI, 15.8%-26.1%). Among 22 patients who have cervical stromal invasion, the details of abnormal Pap smear results included adenocarcinoma (n=10), atypical glandular cells (n=8), ASC-H (n=2), HSIL (n=1), and ASC-US (n=1). Patients with cervical stromal invasion were more likely to have pre-operative abnormal Pap smears compared to those without, and patients with type II endometrial cancer were more likely to have cervical stromal invasion than those with type I (38.36% vs 20.00%). The risk of cervical stromal invasion was higher among premenopausal compared to postmenopausal patients (Table 1).

Table 2 shows the results of binary logistic regression analysis to determine the correlations between abnormal Pap smear results and risk of cervical stromal invasion. Endometrial cancer patients who had abnormal Pap smears had a significantly higher risk of cervical stromal invasion (adjusted OR 2.65; 95% CI 1.35 to 5.21), as did those patients who were diagnosed with type II endometrial cancer (OR 2.33; 95% CI 1.24 to 4.41). 

The percentage of cervical stromal invasion included 65.2% of patients with type II endometrial cancer and abnormal Pap smear, 44.0% of type I endometrial cancer patients with abnormal Pap smear, and 38.3% of type II endometrial cancer patients without abnormal Pap smear. 

## Discussion

One important issue when managing patients with endometrial cancer is identifying those at greatest risk of cervical stromal invasion. In this study, abnormal Pap smear results were found in 21.0% of patients. Abnormal Pap smear and type II endometrial cancer were associated with an increased risk of cervical stromal invasion. 

The rate of abnormal Pap smear results in endometrial cancer patients varies widely in the previous literature (from 24.7-71.2%) due to differences in criteria used to determine whether smear results were abnormal, differences in cervical smear techniques, and potential recall bias (Nadaf et al., 2017; Milicic et al., 2015; Gu et al., 2001). The rate of abnormal Pap smear results was low in this study due to the fact that we used a conventional technique, that did not include findings of debris in abnormal results, excluded patients with gross cervical lesion and performed blinded pathologic review.

Previous studies have aimed to determine the association between abnormal Pap smear results and cervical stromal invasion. Serdy et al. (2006), for example, found that endometrial cancer patients with cervical involvement had a significantly higher rate of results indicating glandular abnormality o than those without (57.1% vs 35.4%). Our findings were consistent with those reported in the literature, in that patients with endometrial cancer who had abnormal Pap smears were approximately three-times as likely to have cervical stromal invasion (95% CI 1.47 to 5.98; Table 2) compared to those who had normal results.

However, Lai et al. (2015) reported that although cervical involvement was more frequently detected by Pap smears (P-value < 0.05) according univariate analysis, multivariate analysis adjusted by histological type and tumor grading revealed no significant association between cervical involvement and Pap smear results. This may have been due to the small number of participants with cervical involvement in that study. 

Cervical stromal invasion was found in 25.1% of patients in this study. However, recent studies have reported lower prevalence (less than 10%) in endometrial cancer patients (Serdy et al., 2016; Vasconcelos et al., 2007; Kitchener et al., 2009; Mascilini et al., 2013). A possible explanation for this discrepancy are differences with regard to tumor characteristics, especially in the proportion of patients with type II endometrial cancer and inconsistent criteria for diagnosing cervical involvement. Recent studies also found that cervical stromal invasion was more frequently observed in serous or clear-cell endometrium cancer around 28-39% of cases (Tate et al., 2018; Murphy et al., 2003). Our data revealed a higher proportion of patients with type II endometrial cancer (about 30.6%) than in other recent studies nearby 10% (Serdy et al., 2016; Vasconcelos et al., 2007; Kitchener et al., 2009; Mascilini et al., 2013).

The strength of this study is that we adjusted for potential confounding factors, such as type of endometrial cancer, in order to determine whether abnormal Pap smear results were significantly and independently correlated with the risk of cervical stromal invasion. Additionally, this study was conducted in a single center, where the histopathological specimens were able to be reviewed by an expert gynecologic pathologist, and the criteria for evaluating the cervical stromal invasion were standardized. The limitations of this study were that it was a cross-sectional study that did not result in oncological outcomes, no liquid-based cervical cytology was conducted, and there were no available parametrial tissues for evaluating the extent of disease due to the fact that all hysterectomies conducted were simple.

In summary, abnormal Pap smear results within 6 months prior to surgery were associated with a higher probability of cervical stromal invasion in patients with endometrial cancer. In addition, patients whose endometrial tissue was histopathologically determined to be type II had a higher risk of cervical stromal invasion.
